# Facile and Chemically Pure Preparation of YVO_4_:Eu^3+^ Colloid with Novel Nanostructure via Laser Ablation in Water

**DOI:** 10.1038/srep20507

**Published:** 2016-02-04

**Authors:** Haohao Wang, Osamu Odawara, Hiroyuki Wada

**Affiliations:** 1 Interdisciplinary Graduate School of Science and Engineering, Tokyo Institute of Technology, 4259 Nagatsuta-cho, Midori-ku, Yokohama 226-8502 Japan

## Abstract

A YVO_4_:Eu^3+^ colloid with an interesting nanostructure was formed by pulsed laser ablation in deionized water without any additives or surfactants. Analyses of particle morphology, composition and optical properties were accomplished by SEM, TEM, EDS PL and UV-vis. Ovoid-like particles formed by the agglomeration of numerous nanocrystals were observed by SEM and TEM, while EDS with area-mode analysis revealed that the content of dopant ion was well retained within the nanoparticles. In addition, the formation mechanism is deduced and discussed for the first time in this research. The findings of this study could provide new insights into the understanding of laser-induced oxide materials and offer an opportunity for other research groups to pursue red emitting nanophosphors with outstandingly purity.

During the past decade, intensive research has been devoted to exploring rare-earth doped materials because they have various potential applications based on their novel optical properties resulting from their 4f electrons^1^,^2^,^3^. Among a huge number of rare-earth doped materials, YVO_4_:Eu^3+^ is a significant red-emitting phosphor; it is already used widely in color television, high-pressure mercury lamps, and as a scintillator in medical image detectors^4^,^5^ because of its suitable crystal structure and high chemical stability^6^. In particular, YVO_4_:Eu^3+^ nanocrystals have optical properties and low cytotoxicity that give them promise for biological applications^7^, and YVO_4_:Eu^3+^ nanocrystals have already been successfully used for biomolecule detection^8^.

In addition, dramatic efforts also have been dedicated to discovering new methods for the synthesis of a range of inorganic nanocrystals to enhance their current performance in biological applications. The precise manipulation of nanoparticles with well-defined morphologies and tunable sizes remains a challenging research issues. The use of chemical methods with additives is one approach used to achieve this control. For instance, Z. Zhou *et al*.^9^ reported a sol-gel method to tune particle morphology with the help of citric acid and PVP, and X. Wu *et al*.^10^ and C. Li *et al*.^11^ reported chemical approaches to control particle size, structure and shape after adding CTAB and Cit^3+^, respectively. However, all these chemical methods, as far as we know, suffer from difficulty removing organic additives after chemical reactions which would seriously influence any bio-labeling and bio-detector applications.

Laser ablation in liquid (LAL) has attracted more and more attention in recent years, resulting in nanoparticle colloids with outstanding purity produced from a variety of materials^12^,^13^,^14^,^15^,^16^,^17^,^18^,^19^,^20^. For instance, a wide array of functional nanoparticles has been synthesized by LAL from materials including semiconductors^21^,^22^,^23^, metals^24^,^25^,^26^, alloys^27^ and even oxide nanoparticles^28^,^29^. In comparison with conventional chemical methods, the LAL technique has many distinct advantages. First, chemical additives are not required in this process, and thus, the aqueous colloids are 100 percent pure, providing ligand-free nanoparticles. This was also the main purpose and reason we used LAL to prepare our nanocrystals. Second, LAL is a very safe, one-step process. Additional high pressure and high temperature are not needed for the LAL process, which is fast, sufficient and safe, excluding the minimal potential for explosion^30^. Last, low-cost is one important advantage; only pulsed laser equipment is needed.

In this paper, we demonstrated the preparation of chemically pure YVO_4_:Eu^3+^ nanocrystals by laser ablation in water for improved applications, especially in the biological field, and explored the effect of energy density on obtaining nanoparticles. To the best of our knowledge, relevant research regarding laser-induced YVO_4_:Eu^3+^ nanocrystals has not yet been reported. Importantly, the details of the LAL mechanism have not been clearly understood until now; these interesting findings may provide new insights and call more attention to the understanding of synthesizing oxide materials using LAL.

## Methods

### Nanocrystal synthesis

The target was shaped into a pellet with a diameter of 10 mm by pressing the commercially available Y_0.95_VO_4_:0.05Eu^3+^ at 100 MPa for 3 min at room temperature. The pellet was then sintered at 1100 °C for 3 h in air. Next, the resulting pellet was placed on the bottom of a small glass vessel and immersed in 3 ml DI water. The thickness of the water layer above the pellet was approximately 15 mm. Colloidal nanoparticles were synthesized by irradiating the pellet using a Q-switched Nd:YAG pulsed laser (Spectron Laser Systems Ltd., SL8585G) providing 13 ns pulses at a 532 nm wavelength and a repetition rate of 10 Hz. The fluence of the laser was varied in the range of 1.7–8.9 J/(cm^2^·pulse) using a neutral density filter, and the irradiation time was set at 20 min. The colloidal nanoparticles were filtered with a 0.22 μm pore-size filter (Rephile Bioscience. Ltd, RephiQuik Syringe Filter).

### Characterization

Phase analysis of the target and the nanoparticles was performed using an X-ray diffractometer (XRD, Philips, X’Pert-PRO-MRD, Netherlands) with a Cu-kα_1_⁄kα_2_ ratio of 0.514 radiation at 55 kV and 22 mA. All patterns were recorded over the angular range 10 ≤ *2θ*/deg ≤90 with a step size of *2θ* = 0.06 deg. The particle size and morphology were observed using scanning electron microscopy (SEM, Hitachi High-technologies, S-5500, Japan). Transmission electron microscopy (TEM, JEOL, JEM-2010F, Japan) was used to characterize the lattice fringe at high resolution with an acceleration voltage of 200 kV. The optical properties of the colloidal nanoparticles were analyzed by photoluminescence spectrophotometry (PL, Hitachi High-technologies, F-7000, Japan) with a 150 V Xe lamp and UV-vis (Jasco Corporation, V-670, Japan) at room temperature. Elemental analysis of the point-mode was performed by energy-dispersive X-ray spectroscopy (EDS, EDAX, Genesis APEX2 system, U.S.A.) combined with TEM, while area-mode analysis was produced by EDS (Horiba, ENERGY EX-250, Japan). Except for X-ray diffraction analysis, the colloidal nanoparticles used for all characterization throughout the study were filtered with a 0.22 μm pore-size filter.

## Results and Discussion

### Morphology and Size distribution

**Figure 1:**
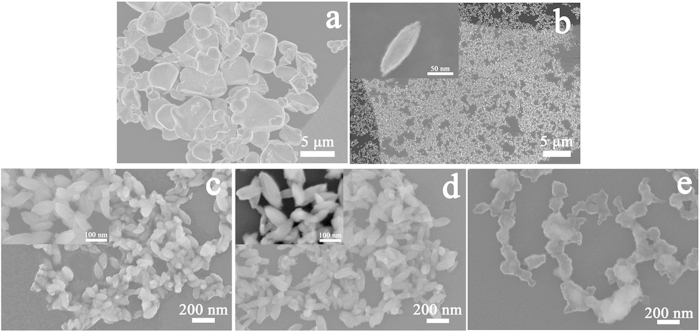
SEM images of (**a**) the targets and (**b**) the nanoparticles obtained at 1.7 J/cm^2^ at low resolution while no filters were applied. Images (**c–e**) were filtered (0.22 μm pore-size) nanoparticles obtained at fluences of 1.7 J/cm^2^, 3.2 J/cm^2^ and 8.9 J/cm^2^ with ×60 k magnification, respectively. Inset image of (**b**) was taken at ×600 k magnification, while insets of (**c,d**) were taken at ×250 k magnification.

Figure 1 shows the SEM images of the target and resulting nanoparticles obtained at different laser fluences. Particles approximately 1–5 μm in size were irregularly shaped, as can clearly be observed. Figure 1(b) shows that nanoparticles fabricated by LAL had an ovoid-like morphology with a diameter of 30–50 nm and a length of 90–120 nm. Notably, all the samples obtained were filtered with a 0.22 μm pore-size filter because of the micrometer-sized particles produced by laser-induced fragmentation^13^,^31^, as shown in supplementary Fig. S1. The effect of fluence on the morphology of nanoparticles was presented in Fig. 1(c–e). Minimal results were detected at lower fluence (1.7 J/cm^2^ and 3.2 J/cm^2^), while nanoparticles obviously started to connect and agglomerate at 8.9 J/cm^2^. We think that the nanoparticle surfaces could have been melted by the higher pulse energy (8.9 J/cm^2^). One reason for this theory is that the melted surfaces would easily attach to neighbouring nanoparticles. Another reason is that the number of nanoparticles obtained at high fluences was larger than at low fluences, which is shown by the UV-vis analysis. Therefore, higher nanoparticle concentration could lead to nanoparticle connection and aggregation. A similar phenomenon was also observed in the research of oxide material performed by T. Nunokawa^32^.

### Structure and Crystallinity

**Figure 2:**
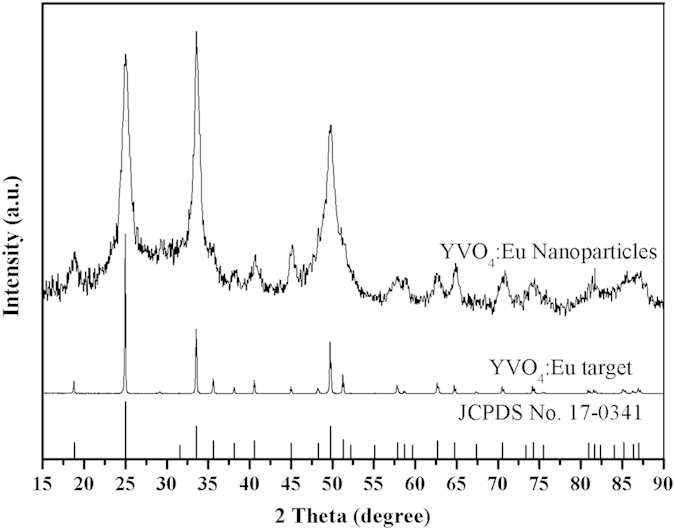
XRD patterns of the YVO_4_:Eu^3+^ target and nanoparticles.

The structure and crystallinity of the YVO_4_:Eu^3+^ target and resulting nanoparticles were analyzed by XRD, as shown in Fig. 2. All diffraction peaks for the target and nanoparticles can be readily indexed as the tetragonal phase of YVO_4_ (JCPDS, No. 17–0341), indicating that all the particles possessed highly crystalline structures without any additional impurity phases. It was also clearly shown that the structure of the laser-generated nanoparticles was identical to that of the target material. In addition, the peaks of the nanoparticles were clearly broadened by their nanosize compared with the peak of the target. However, some peaks, such as those from the (400) plane, did not appear because of the broadened peaks, which overlapped each other. The nanocrystallite size estimated from the full width at half maximum (FWHM) of the (200) peak, according to the Debye-Scherrer formula, revealed that the average YVO_4_:Eu^3+^ crystallite diameter was approximately 8 nm. Note that the nanoparticles used for XRD measurement were not filtered though the 0.22 μm pore-size filters because of the resulting sample volume, which means micron-sized particles were present. Therefore, HR-TEM was performed to reveal the further structure detail of single nanoparticles.

**Figure 3:**
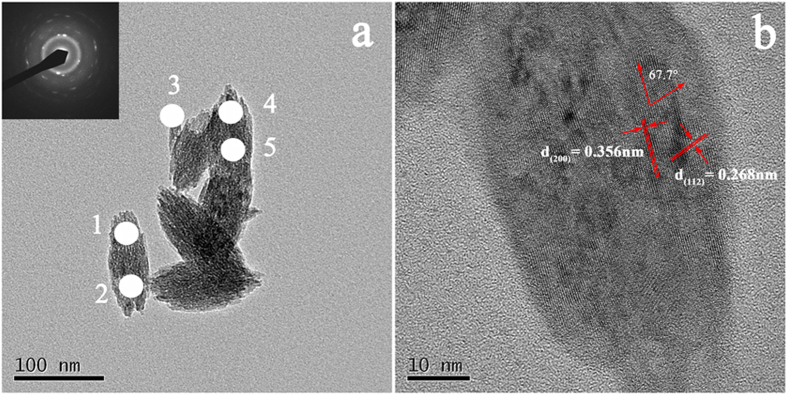
TEM images of the YVO_4_:Eu^3+^ nanoparticles. The inset is the selected area electron diffraction parttern.

Figure 3 displays the HR-TEM results for nanoparticles. Clearly, we can see that the ovoid-like particles are polycrystalline and composed of many smaller nanoparticles. The electron diffraction results for the selected area show the nanoparticles to be highly crystalline. This result also agrees with the crystallite size calculated using Debye-Scherrer equation from the XRD pattern. The measured interplanar distances d_(200)_ of 0.356 nm and d_(112)_ of 0.268 nm are in good agreement with the standard values for YVO_4_ (JCPDS file No. 17–0341). The measured angle, 67.7°, is also in good agreement with the theoretical value.

### Composition analysis

In early works, the laser ablation approach was applied to fabricate metal nanoparticles, though it suffered from the stoichiometry deviations between targets and nanoparticles for alloys, as reported^27^,^33^,^34^. Herein, the quantitative analysis of the dopant ion (Eu^3+^) in a YVO_4_ matrix after laser ablation was assessed by EDS combined with SEM for area-mode. An analysis of 10 different nanoparticle areas and 5 target areas were performed at low resolution for comparison (Supplementary Fig. S2). The ratios of Eu^3+^ in the YVO_4_:Eu^3+^ nanoparticles and target were calculated to be 2.14 ± 0.25% and 2.16 ± 0.16%, respectively, indicating the content of dopant ions was well retained after LAL. Considering that the polycrystalline of ovoid-like nanoparticles and the process of LAL of ultrafast cooling rate and nucleation, EDS with point-mode combined with TEM was performed. Table 1 shows the composition information for 5 points selected at random, as shown in Fig. 3a. The atomic ratio of Eu^3+^ for nanoparticles was 2.8% with a standard deviation of 1.2% in the 5 selected points, whereas the atomic ratio of Eu^3+^ for the target was very stable at 3.0% with a standard deviation of 0.3%.

There are two possible causes of inhomogeneous distribution of Eu^3+^ in the YVO_4_ matrix of the nanoparticles. First, the ovoid-like YVO_4_:Eu^3+^ nanoparticles are polycrystalline, as demonstrated, and it would be possible for different point analyses to contain different numbers of nanocrystallites. There are numerous broken bonds in crystal boundaries, which could cause slight differences in various element contents between the crystal boundary and the inside of the material. Second, because of the complexity and ultrafast nucleation process of LAL, the particle growth rate would be dominated by thermodynamic properties and the density of atoms and clusters of ablated materials^27^,^35^,^36^. We believe that composition segregation might take place, especially because it was also found that the atomic ratio of Eu^3+^ for nanoparticles (4.1%) was sometimes higher than that of target (3.0 ± 0.3%), shown to some extent by EDS point-mode analysis. However, the dynamics and mechanisms of the oxide synthesis by laser ablation in liquid are not yet completely understood. Further investigation is needed in the future.

### Optical properties

**Figure 4:**
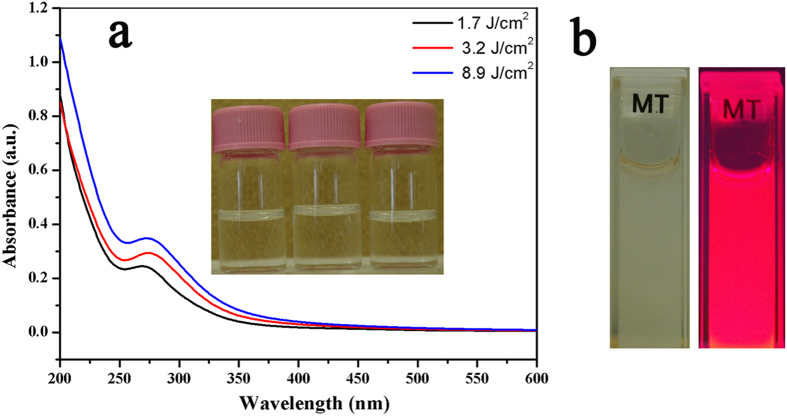
(a) The UV-vis spectra of YVO_4_:Eu^3+^ colloidal nanoparticles at different fluences; (b) YVO_4_:Eu^3+^ (0.5 mg/ml) colloidal nanoparticles obtained at 1.7 J/cm^2^ before(left) and after (right) UV excitation (Ex = 266 nm).

Figure 4a shows the UV-vis absorption spectra of YVO_4_:Eu^3+^ nanoparticles obtained by laser ablation in DI water with different fluences. A strong absorption band peaking at 271 nm, which was attributed to the charge transfer from oxygen ligands to the central vanadium atom in VO_4_^3−^ groups, was observed in three colloidal solutions, agreeing very well with other reports^37^,^38^,^39^. This result also confirms that the absence of by-products after laser ablation in DI water. The insets in Fig. 4a show the transparent colloids obtained from 1.7 to 8.9 J/cm^2^ after 0.22 μm pore-size filtration. In addition, the absorption intensity increased along with the higher fluence, which could be ascribed to the higher productivity attained using higher fluence. Figure 4b shows a luminescent picture of YVO_4_:Eu^3+^ nanoparticles at a 0.5 mg/ml concentration excited by 266 nm UV light; these were obtained by performing 6 iterations (20 min for 1 iteration) of laser ablation at 1.7 J/cm^2^ in DI water.

**Figure 5:**
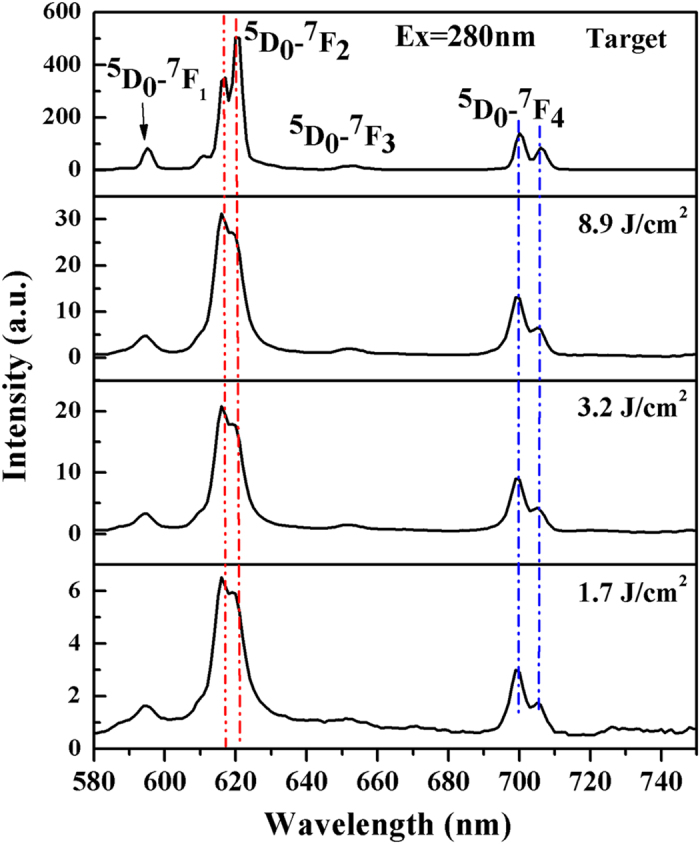
Photoluminescence spectra of YVO_4_:Eu^3+^ colloidal nanoparticles at different fluences (Ex = 280 nm).

The PL spectra of both the target and the YVO_4_:Eu^3+^ colloidal nanoparticles are presented in Fig. 5. As can clearly be observed for both target and nanoparticles, the spectra are dominated by the emission from the europium ions, and mainly the ^5^D_0_-^7^F_2,4_ (electric-dipole transitions), as a consequence of the absence of an inversion symmetry of europium site (*D*_*2d*_ symmetry)^37^,^40^. Other peaks, such as ^5^D_0_-^7^F_1,3_ magnetic dipole transitions, are also observed in nanoparticle samples. However, there is one significant difference, that the ratios of I_615nm_/I_619nm_ and I_700nm_/I_706nm_ that belong to ^5^D_0_-^7^F_2,4_ changed from 0.70 to 1.15 and 1.65 to 2.02, respectively, indicating variations in the symmetry around the europium ions. The symmetry variations further influence the crystal fields around europium ions. It is possible that particle size reduction down to the nanometer range would lead to slight distortions of the crystal lattice due to the increasing effect of the surface. A similar phenomenon was also observed by Wu *et al*.^10^. The PL intensity enhanced by increasing the energy fluence can be ascribe to the higher productivity. This result is confirmed by UV-vis results. Another significant difference is the PL intensity gap between the target and the nanoparticles. An important source of nanoparticle luminescence quenching of is the particle surface, and the coordination of the nanoparticle surface atoms differs from that of the target material because of the broken bonds. Another explanation for the PL intensity gap is that the laser ablation synthesis process was performed in water; therefore, the surface of the nanoparticles could be covered with hydroxyl species, which are the efficient quenchers of europium ions^37^. Note that the quantum efficiency was not measured or discussed here owing to an insufficient number of nanoparticles.

### Possible Mechanism of Crystal Growth

The ovoid-like nanostructure of YVO_4_:Eu^3+^ polycrystals may be explained as follows. “Orientated attachment” proposed by Penn *et al*.^41^ was considered the main path of crystal growth in our case. In this mechanism, the large particles are grown from small primary nanoparticles through an orientated attachment process, in which the adjacent nanoparticles are self-assembled by sharing a common crystallographic orientation, and the overall energy of the system is reduced by the combination of these particles at a planar interface^41^,^42^,^43^,^44^,^45^,^46^. In the resulting larger particles, the crystalline lattice planes of each nanocrystallite are almost perfectly aligned; dislocations at the contact areas between the adjacent particles lead to defects in the final form of the bulk crystals. In addition, it has been reported that the sonochemical method induced the spindle-like morphology of the YVO_4_:Eu^3+^^47^ and the PbWO_4_ polycrystals^48^ using ultrasound irradiation.

**Figure 6:**
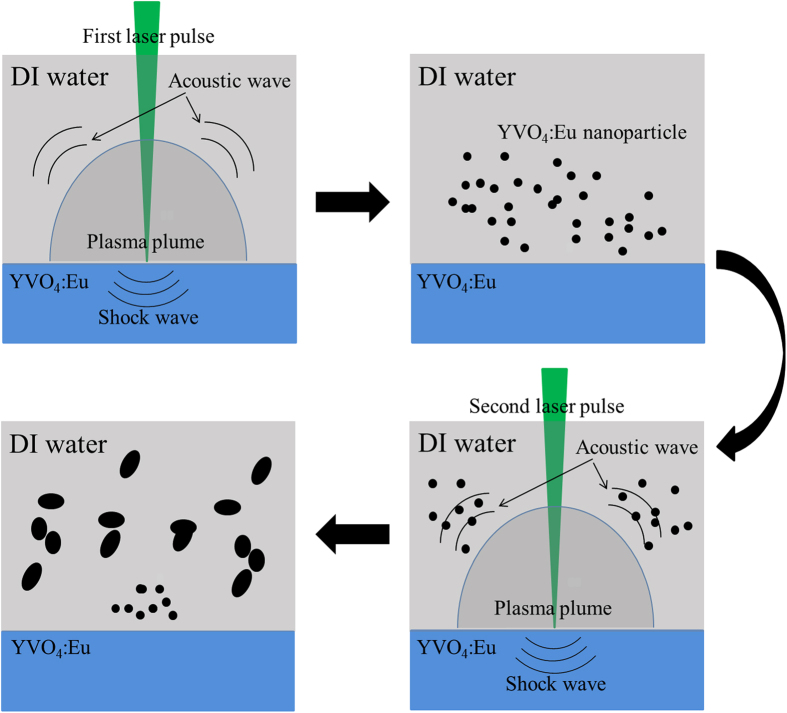
Proposed forming mechanism schematic illustration of the YVO_4_:Eu^3+^ ovoid-like nanostructure upon laser ablation in DI water.

As classical nanoparticle nucleation and growth are described^14^,^30^,^49^, when the very first pulsed laser beam irradiates the target surface in an aqueous environment, a great number of species form in the plasma plume, and the large initial kinetic energy ejects them from the solid target surface to form a dense region in the vicinity of the solid-liquid interface due to the confinement effect of liquid. The liquid limits plasma plume expansion to form an adiabatic region, as the species are confined in the liquid. During this process, acoustic waves are created at supersonic velocity, inducing an extra pressure in the plasma plume. Furthermore, the pressure leads to a temperature increase in the plasma plume. Therefore, a plasma plume state with higher temperature, higher pressure, and higher density is created. The quenching time of the plasma plume in liquid is so short that the nanocrystals are created while the temperature decrease to the phase-transition. Therefore, the formation of the YVO_4_:Eu^3+^ nanocrystallites could be similar to the process of nanocrystal formation by LAL described above. Figure 6 shows schematic diagrams of the formation mechanism of the YVO_4_:Eu^3+^ ovoid-like polycrystalline. The first two images depict a plasma plume composed of the Y, V, and O species with high temperature, high pressure and high density forming and an acoustic wave in water being produced at the same time. YVO_4_:Eu^3+^ nanocrystals were obtained during ultrafast temperature decrese to phase-transition. After that, nanoparticles obatined from the first laser pulse grew and assembled with each other to form ovoid-like particles with the help of the next acoustic wave, which could be attributed to the “orientated attachment”, as shown in the 3^rd^ and 4^th^ images of Fig. 6. Note that these results agree very well with the results from sonochemical approach, from which the spindle nanoparticles were obtained^47^. Furthermore, the nanoparticle HR-TEM result of shows that the ovoid-like particles are composed of numbers of small nanocrystallites via a common crystallographic orientation, as shown in Fig. 3. This result also confirmed our supposition. In fact, Lin^50^ *et al*. also reported the fabrication of CuO nanoparticles by LAL based on the mechanism of “orientated attachment”. Interestingly, in their case spindle-like CuO could be formed by applying an electrical field during LAL process. However, the rod-like nanostructures of CuO were obtained without an electrical field, which led to results different from ours. This may explained by the different crystal growth behaviors of CuO and YVO_4_. Moreover, the further detailed discussion of nanostructure growth kinetics and synthesis upon LAL apporach needs thorough investigation in the future.

## Conclusions

In conclusion, ligand-free YVO_4_:Eu^3+^ colloidal nanocrystals were synthesized by laser ablation in deionized water. XRD, TEM and SEM analysis confirmed that the nanoparticles possessed an ovoid-like shape with high crystallinity in the pure phase. Composition studies using EDS area-mode of the target and nanoparticles showed that the content of dopant ions (Eu^3+^) was well retained after LAL, and composition segregation might take place, as analyzed by point-mode results. Productivity and optical purity were examined using UV-vis, and the emission ratio of I_615nm_/I_619nm_ and I_700nm_/I_706nm_ for nanoparticles changed from 0.70 to 1.15 and from 1.65 to 2.02, respectively, indicating variations in the symmetry around europium ions. The mechanism of the formation of the ovoid-like shape was explained by “orientated attachment” with the assistance of the acoustic wave generated by laser ablation in denionized water.

Continued studies of the YVO_4_:Eu^3+^ nanoparticles are underway to characterize optical properties, such as fluorescence lifetime and quantum efficiency, as well as to investigate potential applications.

## Additional Information

**How to cite this article**: Wang, H. *et al*. Facile and Chemically Pure Preparation of YVO4:Eu^3+^ Colloid with Novel Nanostructure via Laser Ablation in Water. *Sci. Rep.* **6**, 20507; doi: 10.1038/srep20507 (2016).

## Supplementary Material

Supplementary Information

## Figures and Tables

**Table 1:** Atomic ratios of Eu^3+^ in YVO_4_ matrix calculated from EDS point-mode analysis of the targets and the nanoparticles.

EDS (Point-mode): Composition in Atom%	
Area	Eu^3+^
1	2.4
2	2.4
3	3.9
4	1.2
5	4.1
Average	2.8 ± 1.1
Target (Average of 5 particles)	3.0 ± 0.3

## References

[b1] Carlos L., Sá Ferreira R., Pereira R., Assuncao M. & de Zea Bermudez V. White-light emission of amine-functionalized organic/inorganic hybrids: emitting centers and recombination mechanisms. J Phys Chem B 108, 14924–14932 (2004).

[b2] Xu Z. . Ln^3+^ (Ln = Eu, Dy, Sm, and Er) ion-doped YVO_4_ nano/microcrystals with multiform morphologies: hydrothermal synthesis, growing mechanism, and luminescent properties. Inorg Chem 49, 6706–6715 (2010). 20560538 10.1021/ic100953m

[b3] Carlos L., de Zea Bermudez V., Sá Ferreira R., Marques L. & Assunção M. Sol-gel derived urea cross-linked organically modified silicates. 2. Blue-light emission. Chem Mater 11, 581–588 (1999).

[b4] Brecher C., Samelson H., Lempicki A., Riley R. & Peters T. Polarized Spectra and crystal-field parameters of Eu^3+^ in YVO_4_. Physical Review 155, 178 (1967).

[b5] Venikouas G. E. & Powell R. C. Laser time-resolved spectroscopy: Investigations of energy transfer in Eu^3+^ and Er^3+^ doped YVO_4_. J Lumin 16, 29–45 (1978).

[b6] Faria S. & Mehalchick E. YVO_4_:Eu, Tb—An Efficient high pressure mercury vapor lamp phosphor. J Electrochem Soc 121, 305–307 (1974).

[b7] Shen J., Sun L.-D. & Yan C.-H. Luminescent rare earth nanomaterials for bioprobe applications. Dalton Trans, 5687–5697 (2008). 18941653 10.1039/b805306e

[b8] Casanova D. . Single europium-doped nanoparticles measure temporal pattern of reactive oxygen species production inside cells. Nature Nanotechnology 4, 581–585 (2009). 10.1038/nnano.2009.200 19734931

[b9] Hou Z. . Preparation and luminescence properties of YVO_4_: Ln and Y(V, P)O_4_: Ln (Ln = Eu^3+^, Sm^3+^, Dy^3+^) nanofibers and microbelts by sol− gel/electrospinning process. Chem Mater 20, 6686–6696 (2008).

[b10] Wu X. . Morphological control and luminescent properties of YVO_4_:Eu nanocrystals. J Phys Chem B 110, 15791–15796 (2006). 16898727 10.1021/jp060527j

[b11] Li C. . Controlled synthesis of Ln^3+^ (Ln = Tb, Eu, Dy) and V^5+^ ion-doped YPO_4_ nano-/microstructures with tunable luminescent colors. Chem Mater 21, 4598–4607 (2009).

[b12] Dahl J. A., Maddux B. L. & Hutchison J. E. Toward greener nanosynthesis. Chem Rev 107, 2228–2269 (2007). 17564480 10.1021/cr050943k

[b13] Sajti C. L., Sattari R., Chichkov B. N. & Barcikowski S. Gram scale synthesis of pure ceramic nanoparticles by laser ablation in liquid. J Phys Chem C 114, 2421–2427 (2010).

[b14] Liu P., Cao Y., Cui H., Chen X. & Yang G. Micro-and nanocubes of silicon with zinc-blende structure. Chem Mater 20, 494–502 (2007).

[b15] Liu P., Cao Y., Wang C., Chen X. & Yang G. Micro-and nanocubes of carbon with C8-like and blue luminescence. Nano Lett 8, 2570–2575 (2008). 18651780 10.1021/nl801392v

[b16] Liu P., Cui H., Wang C. & Yang G. From nanocrystal synthesis to functional nanostructure fabrication: laser ablation in liquid. Phys Chem Chem Phys 12, 3942–3952 (2010). 20379485 10.1039/b918759f

[b17] Wang J., Zhang C., Zhong X. & Yang G. Cubic and hexagonal structures of diamond nanocrystals formed upon pulsed laser induced liquid–solid interfacial reaction. Chem Phys Lett 361, 86–90 (2002).

[b18] Yan J. . Magnetically induced forward scattering at visible wavelengths in silicon nanosphere oligomers. Nature communications 6 (2015), doi: 10.1038/ncomms8042. PMC4432586 25940445

[b19] Yang G. & Wang J. Carbon nitride nanocrystals having cubic structure using pulsed laser induced liquid–solid interfacial reaction. Applied Physics A 71, 343–344 (2000).

[b20] Yang G.-W., Wang J.-B. & Liu Q.-X. Preparation of nano-crystalline diamonds using pulsed laser induced reactive quenching. J Phys-Condens Mat 10, 7923 (1998).

[b21] Sajti C. L., Giorgio S., Khodorkovsky V. & Marine W. Femtosecond laser synthesized nanohybrid materials for bioapplications. Appl Surf Sci 253, 8111–8114 (2007).

[b22] Usui H., Shimizu Y., Sasaki T. & Koshizaki N. Photoluminescence of ZnO nanoparticles prepared by laser ablation in different surfactant solutions. J Phys Chem B 109, 120–124 (2005). 16850993 10.1021/jp046747j

[b23] Anikin K. . Formation of ZnSe and CdS quantum dots via laser ablation in liquids. Chem Phys Lett 366, 357–360 (2002).

[b24] Simakin A., Voronov V., Shafeev G., Brayner R. & Bozon-Verduraz F. Nanodisks of Au and Ag produced by laser ablation in liquid environment. Chem Phys Lett 348, 182–186 (2001).

[b25] Compagnini G., Scalisi A. & Puglisi O. Production of gold nanoparticles by laser ablation in liquid alkanes. J Appl Phys 94, 7874–7877 (2003).

[b26] Truong S. L. . Generation of Ag nanospikes via laser ablation in liquid environment and their activity in SERS of organic molecules. Appl Phys A 89, 373–376 (2007).

[b27] Jakobi J. . Stoichiometry of alloy nanoparticles from laser ablation of PtIr in acetone and their electrophoretic deposition on PtIr electrodes. Nanotechnology 22, 145601 (2011). 21346297 10.1088/0957-4484/22/14/145601

[b28] Amans D. . Synthesis of Oxide nanoparticles by pulsed laser ablation in liquids containing a complexing molecule: Impact on size distributions and prepared phases. J Phys Chem C 115, 5131–5139 (2011).

[b29] Ledoux G., Amans D., Dujardin C. & Masenelli-Varlot K. Facile and rapid synthesis of highly luminescent nanoparticles via pulsed laser ablation in liquid. Nanotechnology 20, 445605 (2009). 19809121 10.1088/0957-4484/20/44/445605

[b30] Yang G. Laser ablation in liquids: applications in the synthesis of nanocrystals. Prog Mater Sci 52, 648–698 (2007).

[b31] A-Mamun S. A. & Ishigaki T. Influence of hydrogen peroxide addition on photoluminescence of Y_2_O_3_: Eu^3+^ nanophosphors prepared by laser ablation in water. J Am Ceram Soc 97, 1083–1090 (2014).

[b32] Nunokawa T. . Preparation of Y_2_ O_3_: Er, Yb nanoparticles by laser ablation in liquid. Appl Surf Sci 261, 118–122 (2012).

[b33] Abdelsayed V., Glaspell G., Nguyen M., Howe J. M. & El-Shall M. S. Laser synthesis of bimetallic nanoalloys in the vapor and liquid phases and the magnetic properties of PdM and PtM nanoparticles (M = Fe, Co and Ni). Faraday Discuss 138, 163–180 (2008). 18447015 10.1039/b706067j

[b34] Koch J., Von Bohlen A., Hergenröder R. & Niemax K. Particle size distributions and compositions of aerosols produced by near-IR femto-and nanosecond laser ablation of brass. J Anal At Spectrom 19, 267–272 (2004).

[b35] Mafuné F., Kohno J.-y., Takeda Y., Kondow T. & Sawabe H. Formation and size control of silver nanoparticles by laser ablation in aqueous solution. J Phys Chem B 104, 9111–9117 (2000).

[b36] Mafuné F., Kohno J.-y., Takeda Y. & Kondow T. Formation of stable platinum nanoparticles by laser ablation in water. J Phys Chem B 107, 4218–4223 (2003).

[b37] Huignard A., Buissette V., Franville A.-C., Gacoin T. & Boilot J.-P. Emission processes in YVO_4_:Eu nanoparticles. J Phys Chem B 107, 6754–6759 (2003).

[b38] Riwotzki K. & Haase M. Wet-chemical synthesis of doped colloidal nanoparticles: YVO_4_: Ln (Ln = Eu, Sm, Dy). J Phys Chem B 102, 10129–10135 (1998).

[b39] Riwotzki K. & Haase M. Colloidal YVO_4_:Eu and YP_0.95_V_0.05_O_4_:Eu nanoparticles: Luminescence and energy transfer processes. J Phys Chem B 105, 12709–12713 (2001).

[b40] Mialon G., Turkcan S., Alexandrou A., Gacoin T. & Boilot J.-P. New insights into size effects in luminescent oxide nanocrystals. J Phys Chem C 113, 18699–18706 (2009).

[b41] Penn R. L. & Banfield J. F. Imperfect oriented attachment: dislocation generation in defect-free nanocrystals. Science 281, 969–971 (1998). 9703506 10.1126/science.281.5379.969

[b42] Zhang Q., Liu S.-J. & Yu S.-H. Recent advances in oriented attachment growth and synthesis of functional materials: concept, evidence, mechanism, and future. J Mater Chem 19, 191–207 (2009).

[b43] Pradhan N., Xu H. & Peng X. Colloidal CdSe quantum wires by oriented attachment. Nano Lett 6, 720–724 (2006). 16608271 10.1021/nl052497m

[b44] Zhang Z.-p. . Three-dimensionally oriented aggregation of a few hundred nanoparticles into monocrystalline architectures. Adv Mater 17, 42–47 (2005).

[b45] Zuo F., Yan S., Zhang B., Zhao Y. & Xie Y. l-Cysteine-assisted synthesis of PbS nanocube-based pagoda-like hierarchical architectures. J Phys Chem C 112, 2831–2835 (2008).

[b46] Tang Z., Kotov N. A. & Giersig M. Spontaneous organization of single CdTe nanoparticles into luminescent nanowires. Science 297, 237–240 (2002). 12114622 10.1126/science.1072086

[b47] Zhu L. . Sonochemical synthesis and photoluminescent property of YVO_4_:Eu nanocrystals. Nanotechnology 18, 055604 (2007).

[b48] Geng J., Zhu J.-J. & Chen H.-Y. Sonochemical preparation of luminescent PbWO_4_ nanocrystals with morphology evolution. Cryst Growth Des 6, 321–326 (2006).

[b49] Wang C., Liu P., Cui H. & Yang G. Nucleation and growth kinetics of nanocrystals formed upon pulsed-laser ablation in liquid. Appl Phys Lett 87, 201913 (2005).

[b50] Lin X., Liu P., Yu J. & Yang G. Synthesis of CuO nanocrystals and sequential assembly of nanostructures with shape-dependent optical absorption upon laser ablation in liquid. J Phys Chem C 113, 17543–17547 (2009).

