# Supplementary material for: Facile and Chemically Pure Preparation of YVO4:Eu3+ Colloid with Novel Nanostructure via Laser Ablation in Water
Source: Sci Rep. 2016 Feb 4;6:20507. doi: 10.1038/srep20507 (PMC4740808; doi:10.1038/srep20507)
Supplement: Supplementary Information [file srep20507-s1.pdf]

Supplementary Information

Facile and Chemically Pure Preparation of $YVO_4:Eu^{3+}$ Colloid with Novel Nanostructure via Laser Ablation in Water

Haohao Wang *†, Osamu Odawara †, Hiroyuki Wada †

† Interdisciplinary Graduate School of Science and Engineering, Tokyo Institute of Technology, 4259 Nagatsuta-cho, Midori-ku, Yokohama 226-8502 Japan

* Corresponding authors

Haohao Wang; E-mail address: haohao.w.aa@m.titech.ac.jp; Tel/Fax: +81-45-924-5567

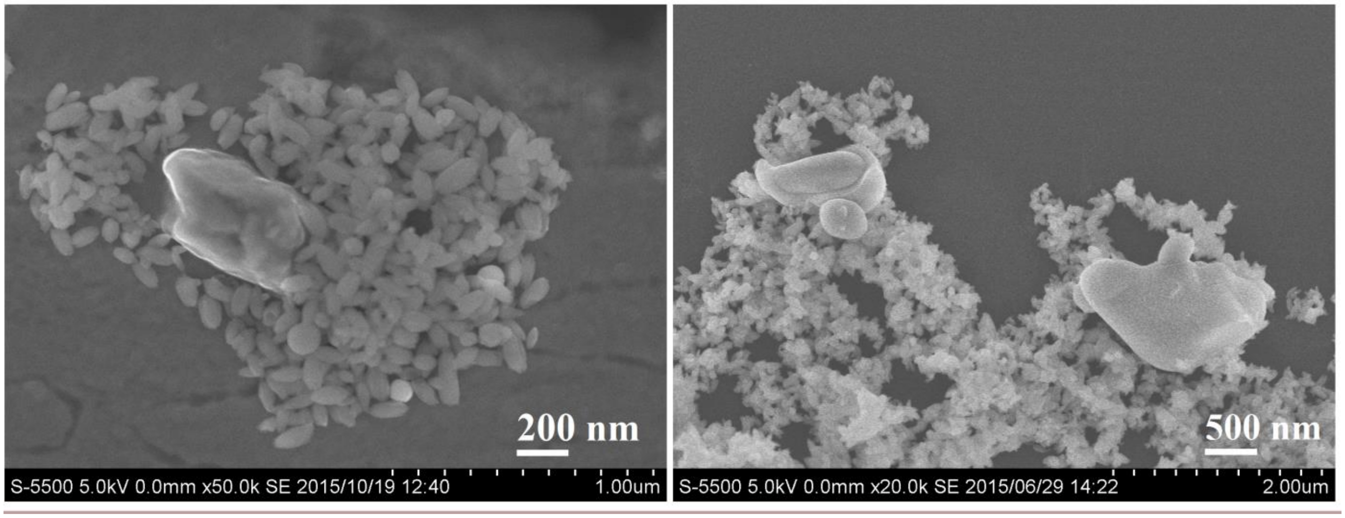

Supplementary Figure S1. Laser-induced micron-sized particles without using 0.22μm pore size filter.

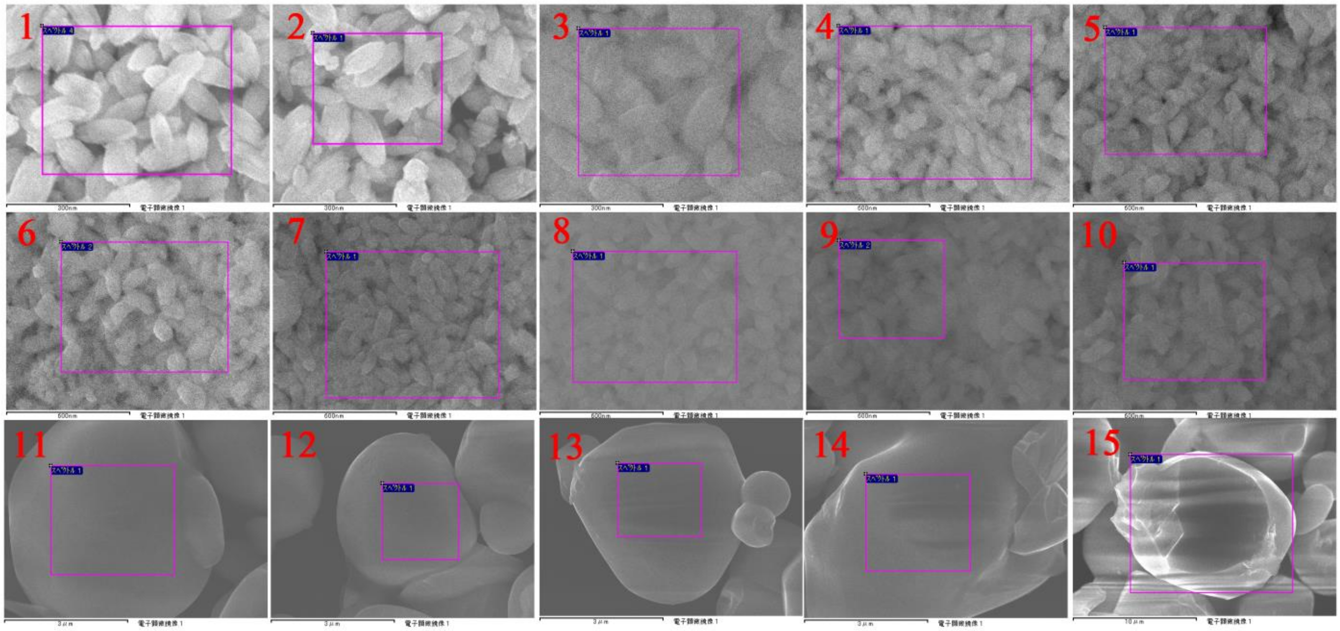

Supplementary Figure S2. (1-10) SEM images of nanoparticles and (11-15) that of target with EDS-area analysis (purple square).
